# La tuberculose extra-ganglionnaire primitive de la sphère ORL: à propos de 15 cas

**DOI:** 10.11604/pamj.2014.19.179.4597

**Published:** 2014-10-21

**Authors:** Mohamed Mliha Touati, Youssef Darouassi, Mehdi Chihani, Mohammed Lakouichmi, Khalid Tourabi, Haddou Ammar, Brahim Bouaity

**Affiliations:** 1 Service d'Oto-rhino-laryngologie et Chirurgie Cervico-Faciale, Hôpital Militaire Avicenne, Marrakech, Maroc; 2 Service de Chirurgie Maxillo-Faciale et Plastique, Hôpital Militaire Avicenne, Marrakech, Maroc

**Keywords:** Tuberculose, sphère ORL, extra-ganglionnaire, Tuberculosis, ENT, extranodal

## Abstract

Les localisations ORL extra ganglionnaires de la tuberculose sont rares. La symptomatologie clinique ainsi que les examens paracliniques sont souvent trompeurs,posant ainsi le problème de diagnostic différentiel avec la pathologie tumorale. Nous rapportons 15 cas de localisations extra ganglionnaires de tuberculose, colligés au service ORL et CCF de l'Hopital Militaire Avicenne de Marrakech colligés entre 2009 et 2013. L’âge moyen de nos patients est de 33 ans. L’étude topographique a montré 6 cas au niveau du cavum, un cas de miliaire tuberculeuse pharyngée, 4 cas laryngés; 2 localisations auriculaires; 1 parotidienne et 1 localisation sous maxillaire. Le diagnostic était anatomopathologiquedans tous les cas. Tous nos patients ont reçu un traitement antituberculeux avec une bonne évolution. Mots-clés: Tuberculose, amygdale, rhinopharynx, larynx, glandes salivaires,Oreille moyenne.

## Introduction

La tuberculose est l'une des maladies infectieuses les plus répandues dans le monde. Au Maroc cette affection sévit encore à l’état endémique et constitue un problème de santé publique. Les localisations ORL sont dominé par l'atteinte ganglionnaire, néanmoins les atteinte extra-ganglionnaire ne sont pas exceptionnelle; il s'agit d'une éventualité relativement rare, de traduction polymorphe et de localisations diverses qui pose parfois des difficultés diagnostiques [1]. Notre travail a pour objectif de mettre le point sur les aspects épidémiologiques, diagnostiques et thérapeutiques de cette pathologie.

## Méthodes

C'est une étude rétrospective effectuée au service d'ORL et CCF de l'Hôpital Militaire Avicenne de Marrakech portant sur 15 cas de tuberculose extraganglionnaire de la sphère ORL colligés durant un période de 6 ans allant de 2009 à 2013. Nous avons analysé les caractéristiques épidémiologiques, les aspects diagnostiques et les modalités thérapeutiques pour chaque localisation.

## Résultats

**Figure 1:**
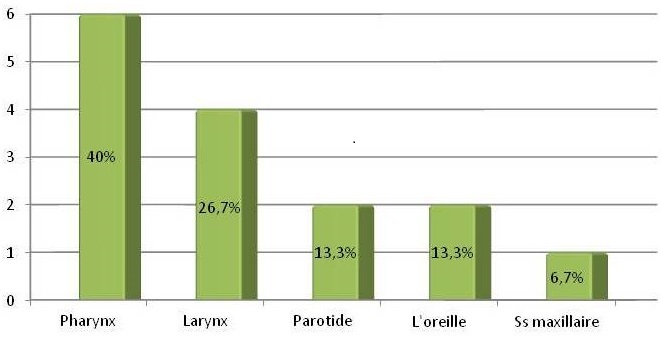
Répartition topographique de la tuberculose extra-ganglionnaire de la sphère ORL des 15 cas étudiés

L’âge moyen de nos patients était de 33 ans, avec des extrêmes de 18 et 70 ans. Il s'agissait de 10 hommes et 5 femmes La notion de contage tuberculeux a été retrouvée chez 2 patients. Le délai moyen entre l'apparition de la symptomatologie et la prise en charge: 2 et 12 mois (moyenne de 4mois). 11 patients soit 73,3% des cas, sont vaccinés contre la tuberculose. Alors que chez 4 patients, la vaccination par le BCG n'est pas précisée. La répartition topographique a montré 11 localisations au niveau des voies aérodigestives supérieures dont 6 cas au niveau du cavum, un cas de miliaire tuberculeuse pharyngée, 4 cas laryngées; 2 localisations auriculaires; 1 parotidienne et 1 localisation sous maxillaire (Figure 1). Le début de la maladie était progressif chez la majorité des patients, aucun patient n'a présenté de signes en faveur d'un déficit immunitaire, dans la majorité des cas l’état général était conservé.

**Pour la localisation cavaire:** le tableau clinique a été dominé par l'obstruction nasale et la rhinorrhée purulente retrouvée dans 50% des cas, les adénopathies cervicales retrouvées dans 50% alors que la dysphagie ne fut observée que chez un seul cas. A l'examen clinique la nasofibroscopie a révélé: - un bourgeon tumoral ulcéro-bourgeonnant du cavum chez 2 cas; - une hypertrophie régulière de la muqueuse cavaire chez 2 cas; - un comblement total du cavum chez 1 cas; - un processus tissulaire rose pale comblant le cavum et arrivant au contact des choanes chez 1 cas.

**Pour la localisation laryngée:** le tableau clinique a été dominé par la dysphonie, retrouvée dans tous les cas. Une dyspnée a été retrouvée dans 50% des cas alors que la dysphagie ne fut observée que chez un seul cas.

**Pour la localisation auriculaire:** retrouvée dans 2 cas qui présentaient une otite moyenne chronique polyploïde avec un pus crémeux, rebelles aux traitements habituels.

**Pour la localisation parotidienne:** elle a été révélée par une tuméfaction parotidienne d'installation progressive, indolore, de consistance ferme non inflammatoire et recouverte d'une peau d'aspect normal.

**Figure 2:**
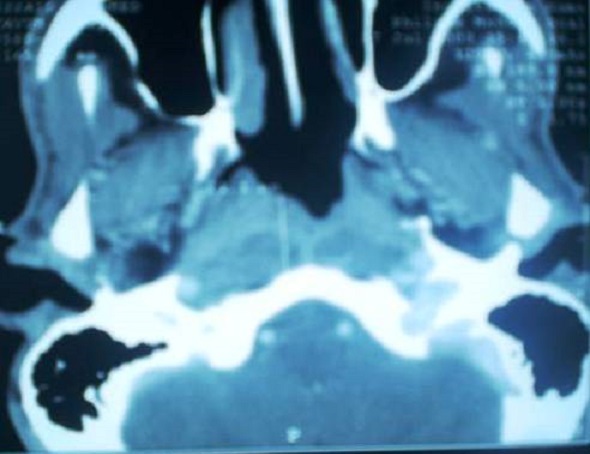
TDM cervicale en coupe axiale montrant un processus tumoral de la paroi postéro latérale droite du rhinopharynx et des adénopathies rétro pharyngées

**Pour la localisation sous maxillaire:** elle s'est révélée par une tuméfaction maxillaire de consistance ferme, non inflammatoire. Sur le plan paraclinique, l'IDR: elle a été positive dans 06 cas, négative dans 07 cas et non faite dans 2 cas. La CRP: accélérée chez 8 cas. La sérologie VIH a été demandée dans 8 cas, elle s'est révélée négative. La radiographie pulmonaire réalisée chez tous nos patients était normale. La tomodensitométrie a été faite chez 12 patients, essentiellement pour les localisations cavaires et pharyngée pour 7patients (Figure 2), laryngée pour 4 patients, et parotidienne pour 1 patient, L’échographie faite dans un cas de la tuberculose sous maxillaire, a montré une masse tissulaire hétérogène avec des contours polylobés. La recherche systématique de BAAR dans les secrétions bronchiques a été négative chez tous les patients. Le diagnostic a été basé sur la confirmation histologique par la mise en évidence des lésions epithélio-giganto-cellulaire avec nécrose caséeuse dans tous les cas.

Le traitement a consisté en un traitement médical de 6 à 9 mois. Le traitement chirurgical a intéressé 2 localisations: parotidienne et auriculaire dans un but diagnostique. L’évolution sous traitement a été satisfaisante, le contrôle après trois mois d′arrêt du traitement a montré une régression des signes cliniques, endoscopiques et radiologiques, les patients sont toujours suivis de façon régulière sans aucune récidive locale avec un recul moyen de 32 mois.

## Discussion

Tous les indices épidémiologiques insistent actuellement sur la recrudescence de la tuberculose dans le monde entier. On estimait à 8,7 millions le nombre de nouveaux cas de tuberculose (dont 13% Co-infectés par le VIH) et 1,4 million de décès [2]. Ceci est dû au nombre croissant de sujets immunodéprimés, ou vivants dans des conditions socio-économiques précaires, et à l’émergence de souches multi-résistantes de bacilles tuberculeux [1]. Les localisations ORL en dehors des atteintes ganglionnaires représentent 1,8% de l'ensemble des localisations tuberculeuses. Dans notre étude, l’âge moyen était de 33 ans. Dans la littérature ce chiffre se rapproche de celui des pays du tiers monde. L’âge moyen en Tunisie est de 34 ans, 32 ans en Tanzanie. Dans les pays économiquement avancés, la moyenne d’âge est plus élevée. En France et aux États-Unis, la moyenne d’âge est de 52 ans. Cependant, ces chiffres n'excluent pas l'atteinte ganglionnaire [3].

Le larynx représente le site le plus affecté par la tuberculose, pouvant atteindre un taux de 46% [1, 4]. Tous les auteurs s'accordent sur la prédominance masculine de cette pathologie, qui semble être favorisée par une intoxication alcoolo- tabagique excessive. Gallas [5] estime que la fréquence de l'intoxication tabagique chez les patients présentant une tuberculose laryngée est de 72,6%, dans notre série, nous avons retrouvé 6 fumeurs soit 60% dont 3 alcoolo-tabagiques. Sur le plan clinique, La dysphonie chronique reste le maitre symptôme de la tuberculose laryngée (76–96% des cas) [6]. Les aspects endoscopiques peuvent prendre plusieurs formes et prêtent à confusion dans les formes ulcèrobourgeonnantes et ulcèroinfiltrantes avec la pathologie maligne, c'est l'examen anatomopathologique avec recherche de BAAR qui permet le diagnostic de tuberculose laryngée et d’éliminer la pathologie néoplasique, tout en sachant que leurs association est possible [4].

La localisation cavaire a été initialement décrite par GRAFF en 1936[7]. Elle touche surtout l'adulte jeune entre 20 et 40 ans, avec une répartition égale pour les deux sexes. Les signes cliniques sont semblables à ceux d′un carcinome nasopharyngé, avec adénopathie cervicale généralement unilatérale, obstruction nasale homolatérale, une épistaxis, une rhinorrhée purulente sale ou bien parfois des signes otologiques secondaires à une otite séromuqueuse. Le diagnostic positif repose l'histologie avec présence de granulomes épithélio-giganto-cellulaires avec nécrose caséeuse. Les signes endoscopiques et radiologiques sont surtout en faveur d′un processus tumoral malin [8], d′où la nécessité de réaliser plusieurs biopsies à endroits différents pour pouvoir éliminer un carcinome nasopharyngé. La tuberculose oropharyngée reste dominée par l'atteinte amygdalienne observée dans 45% des cas [4]. Elle prend volontiers une allure pseudotumorale et est le plus souvent de découverte histopathologique. La symptomatologie est dominée par l'odynophagie et la dysphagie haute. A l'examen, on note généralement la présence d'une ulcération de la muqueuse oropharyngée ou une hypertrophie asymétrique d'une amygdale [9].

La localisation parotidienne de la tuberculose est rare mais reste relativement plus fréquente que celle des autres glandes salivaires. Environ 200 cas ont été rapportés dans la littérature entre 1896 et 1987 [10]. Plus de 90% des cas décrits le sont dans les pays en voie de développement. YUSUFHAN rapporte 6 cas de tuberculose parotidienne lors d'une étude de 216 cas de lésions parotidienne soit 2,8% [11]. Plus récemment en 2012 I. Id Ahmed [11] rapporte 2 cas de tuberculose parotidienne dans une étude de 9 cas soit 22%. L'atteinte est souvent secondaire et exceptionnellement primitive. La tuberculose parotidienne se manifeste généralement sous forme d′une tuméfaction parotidienne unilatérale d′installation progressive, pouvant être diffuse ou nodulaire, réalisant un syndrome pseudotumoral, Les signes généraux d′imprégnation tuberculeuse sont rarement présents [10]. Il faut noter que ni l'examen clinique, ni les examens morphologiques ne permettent d’établir le diagnostic de certitude qui est basé sur la parotidectomie exofaciale avec étude histologique.

L'atteinte de la glande sous maxillaire est exceptionnelle, Le tableau clinique de sous-maxillite tuberculeuse est souvent trompeur et pose un problème diagnostique d′autant qu′il n′existe pas de manière contemporaine d′atteinte pulmonaire [12]. La croissance lente de la tuméfaction fait évoquer d′abord une tumeur de la glande et notamment un adénome pléomorphe.

La tuberculose auriculaire est une entité rare, les aspects cliniques sont très trompeurs, en raison du manque de spécificité et de la chronicité des symptômes et souvent responsables d'un retard diagnostic [13]. C'est souvent le tableau d'une otite moyenne suppurative et rebelle aux traitements habituels. L'aspect de perforations multiples ou bien un Aspect dénudé du promontoire (carie blanche) sont très évocateurs, Parfois le diagnostic est évoqué devant l’échec de plusieurs tympanoplasties et c'est le prélèvement d'un fragment de la muqueuse de la caisse pour examen histologique qui permet le diagnostic [13]. La certitude de la tuberculose en ORL tout siège confondu est histologique et /ou bactériologique [4]. L'aspect histologique est caractéristique devant la constatation d'une nécrose caséeuse associée à un granulome épitheloide et gigantocéllulaire, l'absence de cette nécrose oriente vers les autres pathologies granulomateuses. Le fragment biopsique est mis en culture systématiquement sur milieu de Lowstein afin d'isoler le germe, typer l'espèce et réaliser un antibiogramme très utile dans les formes résistantes ou en cas d'antécédents de traitement anti tuberculeux ou sur un terrain particulier (HIV) [13].

Les symptômes les plus retrouvés ne sont pas spécifiques quelle que soit la localisation ORL. De même, l'imagerie à type d’échographie, de TDM ou encore d'IRM, n'est pas d'un grand secours. L'intradermoréaction à la tuberculine n'est pas toujours positive. Tous les patients n'en ont pas bénéficié car bon nombre d'entre eux étaient des surprises diagnostics.

Cependant, l'histologie peut apporter une preuve formelle dans un délai raisonnable (moins d'une semaine), contrairement à la bactériologie dont le résultat (culture) peut nécessiter plusieurs mois. En effet, elle révèle la présence de foyers épithélio-gigantocellulaires avec nécrose caséeuse. Pour l'ensemble de nos patients,l'histologie étais suffisante pour confirmer le diagnostic positif nous évitant ainsi d'attendre le résultat de la culture et nous permettant de démarrer le traitement rapidement. Lorsque l'analyse microscopique des lames ne retrouve pas d'images de nécrose lors d'une supposée tuberculose nasosinusienne ou nasopharyngée, le diagnostic différentiel se pose avec la sarcoïdose, la granulomatose de Wegener, la syphilis ou la lèpre [13], imposant des techniques plus sophistiquées comme la polymérase Chain réaction (PCR) qui permet de trancher en détectant la mycobactérie tuberculeuse. Au Maroc, pays d'endémie, la tuberculose survient souvent sur des terrains immunocompétents. La sérologie HIV n'est demandée qu'exceptionnellement s'il existe d'autres signes d'appel, 8 de nos patients ont bénéficié de cet examen qui c'est révélé négatif. Si la vaccination BCG est obligatoire au Maroc et si tous les patients en ont bénéficié, cela montre bien que celle-ci ne protège pas totalement, même contre les formes rares de la tuberculose. Le traitement est le plus souvent strictement médical s'appuyant sur les antibacillaires pour une durée minimale de six mois [3, 12]. Dans la prise en charge de cette pathologie la chirurgie n'a qu'une place minime réservée aux formes pseudotumorales en vue d'une confirmation anatomopathologique, l’évolution est presque toujours favorable. Les rares cas d’échec (1%) s'expliquent par l’émergence de souches résistantes aux Antituberculeux [3].

## Conclusion

La tuberculose extra-ganglionnaire de la sphère ORL est une pathologie rare. Cliniquement elle pose des difficultés diagnostiques avec la pathologie néoplasique. Le diagnostic est essentiellement histo-pathologique. Le traitement est basé sur les anti-tuberculeux. L’évolution est généralement favorable.
